# Large graft tectonic penetrating keratoplasty in a case of severe aspergillus keratitis

**DOI:** 10.3205/oc000144

**Published:** 2020-04-02

**Authors:** Albert John Bromeo, Elenor Reina Aquino-Alegre, Ruben Lim Bon Siong

**Affiliations:** 1 Department of Ophthalmology and Visual Sciences, Sentro Oftalmologico Jose Rizal, Philippine General Hospital, University of the Philippines, Manila, Philippines

## Abstract

Penetrating keratoplasty is indicated for cases of severe microbial keratitis, particularly if associated with impending corneal perforation. The case report details a 45-year-old male farmer who consulted for blurring of vision in the left eye after an incident wherein mud was flung onto his eye during farming. He noted eye redness and a growing opacity on his left eye. He was initially treated with topical antimicrobial and corticosteroid medication which did not resolve his symptoms. He presented with a visual acuity of hand movement on the affected eye. Slit lamp examination showed a large protruding mound-like plaque, occupying almost the entire corneal surface of the left eye, with associated scleritis. The ocular ultrasound was unremarkable. The patient was diagnosed with fungal keratitis, which culture from corneal scraping showed to be from an infection with *Aspergillus*. A tectonic penetrating keratoplasty with 360-degree iridectomy, lens extraction, and anterior vitrectomy was immediately done, and a regimen consisting of topical natamycin was started. Despite the severe presentation of the fungal corneal infection, the eye was fortunately salvaged.

## Introduction

Fungal keratitis remains a challenge for ophthalmologists, particularly in developing countries. It has been shown to be more virulent than most cases of bacterial keratitis and often requires penetrating keratoplasty [1]. Clinical signs which help distinguish fungal keratitis from bacterial keratitis include characteristic feathery border of ulcers, presence of ring infiltrates, satellite lesions, and propensity for plaque formation [2]. Severe cases usually result in penetration of the fungi into the anterior chamber, leading to anterior endophthalmitis and eventually endophthalmitis. These cases inevitably have a significantly poorer prognosis [2].

The following case report discusses a case of an unusual presentation of fungal keratitis forming a very large mound-like plaque occupying almost the entire corneal surface.

## Case description

A 45-year-old male farmer consulted with a chief complaint of blurred vision in the left eye. The history started about 2 months prior when mud was flung onto his left eye while farming. He washed his eye with tap water and carried on. About 2 weeks later, he noted development of redness, blurred vision, mild pain, and a small white opacity on the left eye. He consulted with a private physician who assessed him to have an eye infection, and he was started on topical terramycin ointment. Despite treatment, there was persistence of eye redness and enlargement of the whitish opacity. The patient sought a second opinion with another physician, who prescribed him a regimen consisting of tobramycin-dexamethasone drops, again with no improvement in the patient’s symptoms. The patient eventually noted loss of useful vision in his left eye. The persistence of symptoms prompted consult.

**Figure 1:**
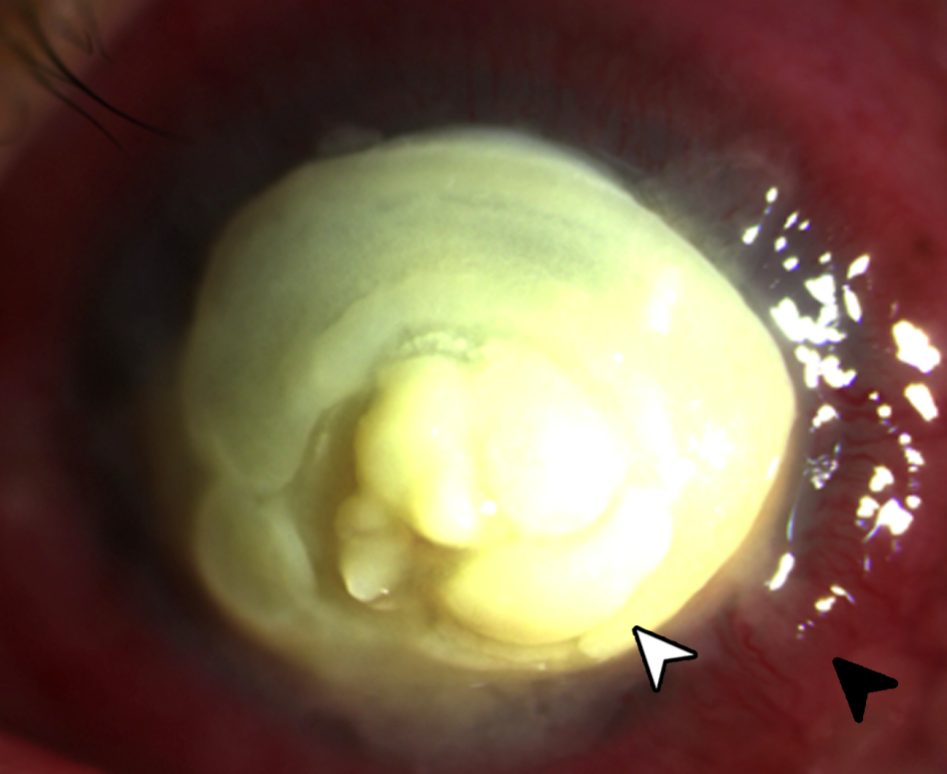
Slit lamp photograph of eye showing a large yellowish plaque (white arrowhead) occupying almost the entire cornea with surrounding scleritis (black arrowhead).

On presentation, the visual acuity of the left eye was hand movement with poor light projection. There was good color perception. A reverse relative afferent pupillary defect was not noted. On slit lamp examination, there was a very thick yellow plaque consisting of a rounded mass projecting above the entire corneal surface, similar to a mound, measuring 9x9.5 mm with a depth of 2 mm at its thickest point located centrally (Figure 1 (Fig. 1)). Despite the large plaque, there seemed to be no signs of prolapse of intraocular contents. There was associated scleritis. The ocular ultrasound showed only few vitreous cellularities. The right was grossly unremarkable with a visual acuity of 20/20.

**Figure 2:**
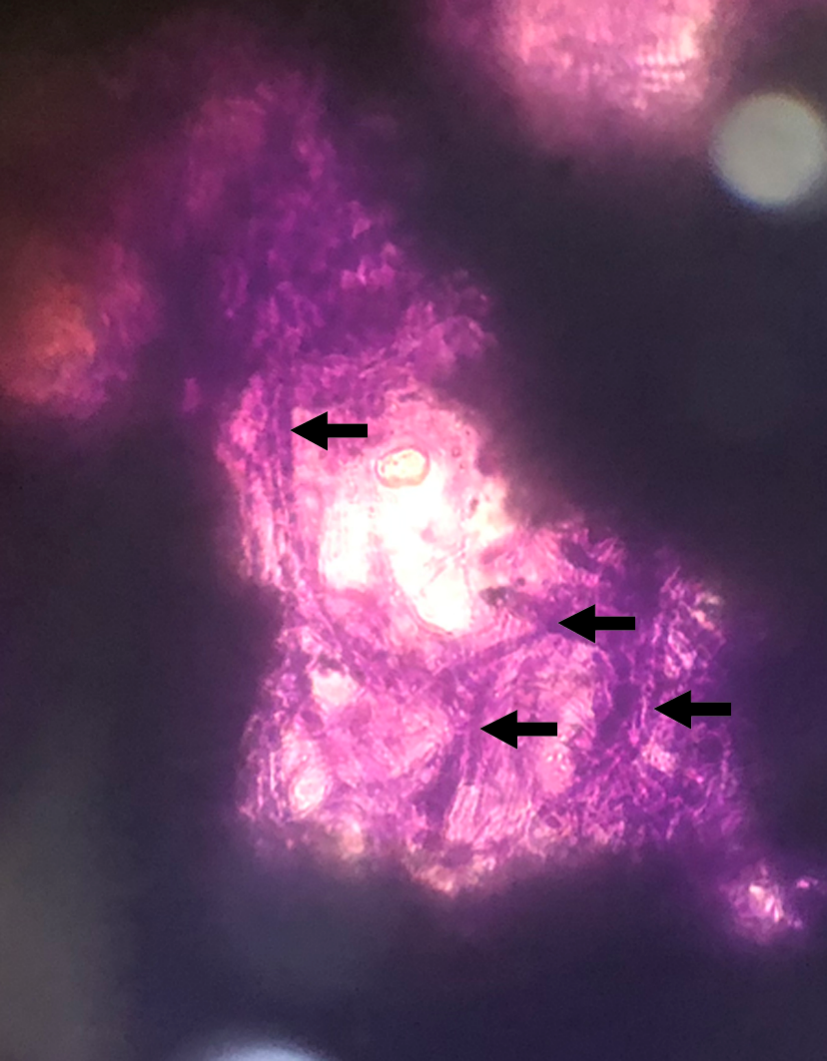
Giemsa stain of scrapings obtained from the corneal plaque showing an extensive amount of hyphal elements (black arrows).

The plaque did not stain with fluorescein, lissamine green, and rose bengal dyes. Careful scraping of the mass revealed it to be semi-solid, albeit very friable in consistency. There was no bleeding on scraping. Giemsa stain of the corneal scraping showed numerous hyphal elements (Figure 2 (Fig. 2)). Culture of the plaque later revealed to be positive for *Aspergillus* sp.

The patient was admitted and initiated on topical natamycin drops, and topical moxifloxacin drops were added as prophylaxis for any superimposed bacterial infection. Topical atropine drops were also started for relief of ciliary pain. The patient was started on oral itraconazole as well.

**Figure 3:**
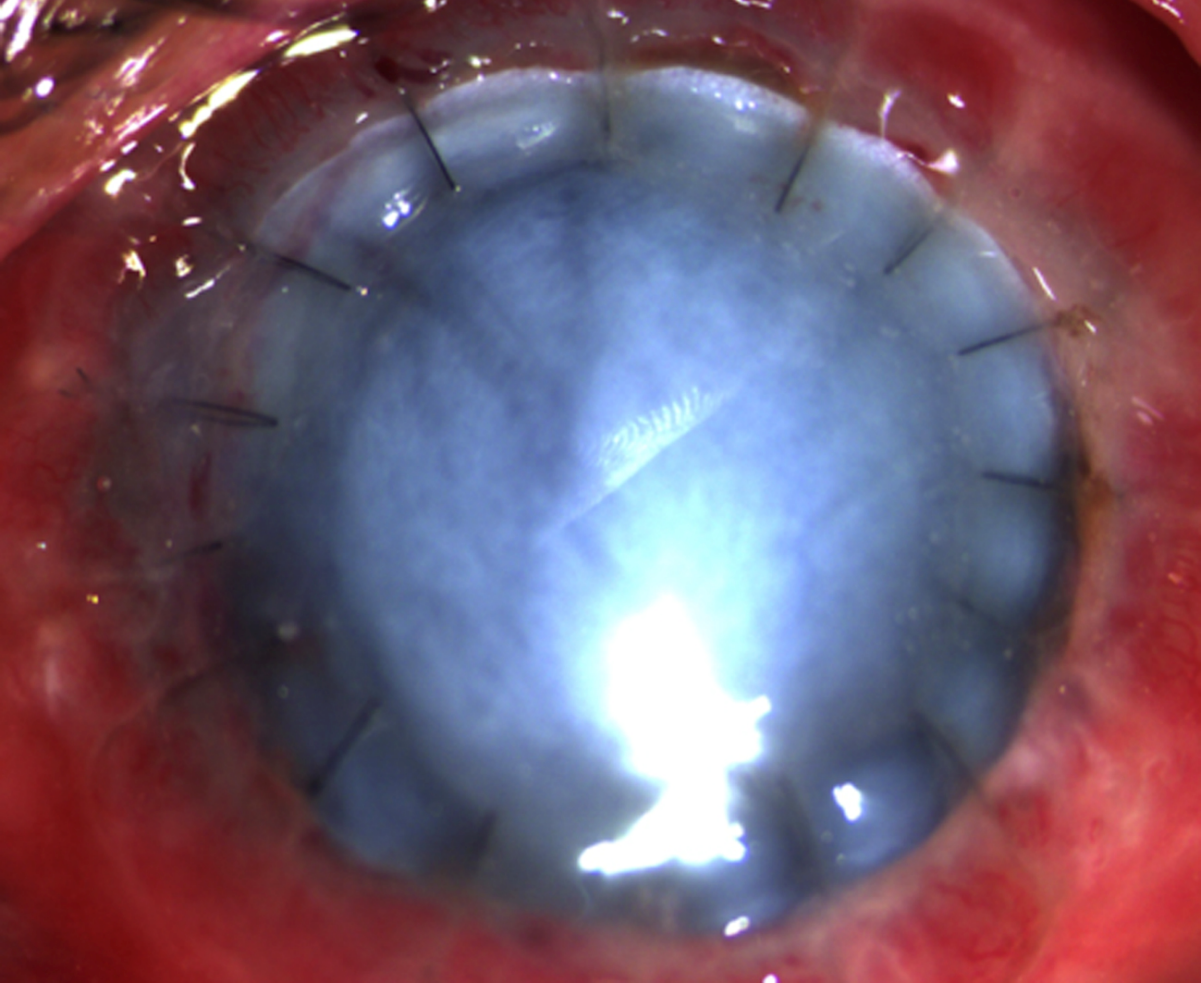
Slit lamp photograph of eye at the first day postoperatively showing a tectonic graft held in place with #16 interrupted nylon 10-0 sutures.

A therapeutic keratoplasty with a tectonic corneal graft was performed under retrobulbar anesthesia. The recipient bed was sized by measuring the fungal plaque and adding a 2 mm border. The corneal graft was sized to a 12 mm diameter, necessitating manual cutting using corneal scissors. After the host cornea was excised, it was revealed that the fungus had invaded the anterior chamber, and the iris had become necrotic. A 360-degree iridectomy was performed. Lens extraction and anterior vitrectomy were done. After reforming the anterior chamber with an ophthalmic viscoelastic device, the tectonic corneal graft was placed and sutured with 16 interrupted nylon 10-0 sutures (Figure 3 (Fig. 3)). The surgery was uncomplicated.

The patient was maintained on a regimen of topical natamyin, topical moxifloxacin, and topical atropine drops for the first 2 weeks after surgery. He also completed a 2-week course of oral itraconazole. The postoperative course was relatively unremarkable with decreasing signs of inflammation and no recurrence of infection. At the 2^n^^d^ week postoperatively, topical prednisolone drops were added. The patient’s vision improved to counting fingers at 6 inches. He was advised that a second procedure consisting of an optical penetrating keratoplasty and secondary intraocular lens implantation as well as possible interventions for secondary glaucoma would be needed in the future.

## Discussion

Fungal keratitis is a fungal infection of the cornea. It is often considered a disease associated with rural farming areas, since it is traditionally associated with a history of trauma with vegetative material. However, contact lens use seems to now be the major risk factor in developed countries [2]. It has been described to occur from infections from both filamentous (*Aspergillus* sp. and *Fusarium* sp.) and yeast (*Candida* sp.) fungi [2].

It remains a challenge for ophthalmologists since initial presentation may mimic bacterial keratitis. Antimicrobial agents used in bacterial keratitis are not effective for fungal keratitis, thus continued fungal growth occurs before the ophthalmologist recognizes failure of response. Some cases may be misdiagnosed as immunologic reactions and treated with corticosteroids, which decrease immune response, thus promoting fungal growth. In addition, since the disease is traditionally associated with farmers living in rural communities, significant delays in diagnosis often occur before appropriate treatment [3].

The case presented showed an unusual growth pattern of *Aspergillus* keratitis wherein the fungal plaque became an exuberant mound-like structure. Plaques associated with fungal keratitis are usually planar, and often still follow the contour of the host cornea [2]. Factors associated with the unusual growth pattern presented in the cases include a significant delay before initiation of appropriate treatment (2 weeks before diagnosis and an additional 6 weeks of inappropriate antimicrobial treatment) and initiation of topical medication containing a corticosteroid component. It is also unusual that despite the exaggerated exophytic growth of the infection, the fungi were only able to invade up to the anterior chamber. Indeed, the patient was fortunate since invasion of the posterior segment will portend a significantly poorer prognosis [1].

A penetrating keratoplasty was done to decrease fungal load for greater efficacy of topical antifungal medications, as well to prevent further growth of the fungi into the globe. In this procedure, the diseased portion of the cornea is excised and replaced with a donor corneal graft. The procedure has been shown to be effective in cases of fungal keratitis [4], [5]. The major risk of performing keratoplasty in cases of fungal keratitis is recurrence of the fungal infection. Thus, a regimen consisting of topical antifungal drops with topical antimicrobrial drops to cover for secondary bacterial infection is continued for a prolonged period of time following successful keratoplasty. Natamycin is frequently used as initial therapy due to its availability. Voriconazole and amphotericin B are also emerging as promising first-line therapy in select cases [2], [5]. In addition, topical corticosteroids are withheld for at least 2 weeks in the early postoperative period [3].

A combination of expedient penetrating keratoplasty and initiation of appropriate culture-guided antifungal medications was able to salvage this patient’s eye, and recovery of useful vision in the future will be possible.

## Conclusion

Fungal keratitis is a fungal infection of the cornea caused by both filamentous and yeast fungi. Risk factors include trauma with vegetative material and contact lens use. It is associated with ulcers with feathery borders, ring infiltrates, plaque formation, and penetration into the anterior chamber. The cornerstone of management of fungal keratitis is initiation of culture-guided antifungal therapy. Penetrating keratoplasty may be needed in severe cases. Corticosteroids should never be given in cases of active fungal keratitis.

## Notes

### Competing interests

The authors declare that they have no competing interests.

### Ethical statement

The case report is a minimal-risk study which was conducted in full compliance with the principles of the Declaration of Helsinki and Good Clinical Practice. All identifying patient information was kept confidential. Informed consent was obtained from the family of the patient.
